# Major incident preparedness and on-site work among Norwegian rescue personnel – a cross-sectional study

**DOI:** 10.1186/1865-1380-5-40

**Published:** 2012-11-07

**Authors:** Sabina Fattah, Andreas J Krüger, Jan Einar Andersen, Trond Vigerust, Marius Rehn

**Affiliations:** 1 Department of Research and Development, Norwegian Air Ambulance Foundation, P.O box 94, Drøbak, 1448, Norway; 2 Anaesthesia and Critical Care Research Group, Faculty of Health Sciences, University of Tromsø, Tromsø, Norway; 3 Department of Anaesthesia and Acute Care, St. Olavs Hospital, Trondheim, Norway; 4 Norwegian Air Ambulance, Lørenskog, Norway; 5 Department of Anaesthesia and Intensive Care, Akershus University Hospital, Lørenskog, Norway

**Keywords:** Emergency medicine, Major incident, Disaster medicine, Cooperation, Preparedness, Accident, Trauma

## Abstract

**Background:**

A major incident has occurred when the number of live casualties, severity, type of incident or location requires extraordinary resources. Major incident management is interdisciplinary and involves triage, treatment and transport of patients. We aimed to investigate experiences within major incident preparedness and management among Norwegian rescue workers.

**Methods:**

A questionnaire was answered by 918 rescue workers across Norway. Questions rated from 1 (doesn’t work) to 7 (works excellently) are presented as median and range.

**Results:**

Health-care personnel constituted 34.1% of the participants, firefighters 54.1% and police 11.8%. Training for major incident response scored 5 (1, 7) among health-care workers and 4 (1, 7) among firefighters and police. Preparedness for major incident response scored 5 (1, 7) for all professions. Interdisciplinary cooperation scored 5 (3, 7) among health-care workers and police and 5 (1, 7) among firefighters. Among health-care workers, 77.5% answered that a system for major-incident triage exists; 56.3% had triage equipment available. The majority – 45.1% of health-care workers, 44.7% of firefighters and 60.4% of police – did not know how long it would take to get emergency stretchers to the scene.

**Conclusions:**

Rescue personnel find major incident preparedness and on-scene multidisciplinary cooperation to function well. Some shortcomings are reported with regard to systems for major incident triage, tagging equipment for triage and knowledge about access to emergency stretchers.

## Background

The remit of the Norwegian rescue services is to undertake immediate efforts to rescue people from death or injury caused by acute emergency situations [1]. Norway has a government-funded emergency medical system coordinated by regional emergency medical control centres (EMCCs), typically co-located with hospital facilities. EMCCs use national dispatch criteria to allocate the correct resource to a medical emergency. This might be a home visit by a general practitioner on call, a direct ambulance dispatch to the patient location or activation of the 18 anaesthesiologist-manned helicopter services (HEMS) placed around Norway [2]. EMCCs work in close cooperation with police and fire and rescue service coordination centres, and the usual approach when facing larger incidents is the triple-activation principle, activating EMS, police and fire and rescue services simultaneously. The backbone of a response to a medical emergency outside the hospital in Norway is activation of ambulance services, and the GP on call in the specific area will have the formal role as medical commander on scene, also in major incidents. Usually this role will be transferred to the air ambulance physician if HEMS is activated.

Pre-hospital emergency medical care in Norway is characterized by time-consuming transport of patients, often under adverse operative conditions [3-5]. On scene, the various emergency services cooperate to perform the key elements of major incident management: leadership, risk management, triage, therapy and transport [6]. The police, the fire department and the health workers all have their pre-determined tasks and are identified by wearing different vests. The police have the general responsibility for coordination and management. The fire department is responsible for extinguishing fires and general rescue efforts (extrication, access, safety), as well as having a particular responsibility for securing any possible hazardous materials. The health services are responsible for treatment and evacuation of patients. During major incidents the cooperating emergency services communicate at the scene using a common VHF radio channel, while EMCCs and regional communication centrals for police in turn are responsible for activation of resources requested from scene.

Accidents are the leading cause of death among persons under 45 years of age in Norway, resulting in approximately 1,800 fatalities each year [7]. A major incident has occurred when the number of persons involved, the type of incident and the location of the incident require extraordinary rescue efforts [6]. Since the capacity of the rescue services varies from one location to another, an accident that outstrips the available capacity in rural areas may be manageable for services in urban areas where the access to resources is better [6]. No national quality measure on major incident management exists, but emergency services aim for an early activation of sufficient personnel, rapid access to a secured scene and efficient patient evacuation to the appropriate facility.

According to national statistics, a total of 103 major incidents occurred in Norway during the period from 1970 through 2001. These incidents include the train collision at Tretten in 1975, the capsizing of the Alexander L. Kielland oilrig in 1980, the avalanche disaster in Vassdalen in 1986, the fire on board the Scandinavian Star in 1990 and the plane crash at Operafjellet on Svalbard in 1996 [8].

The interdisciplinary nature of the Norwegian rescue services was evident during the Åsta accident on 4 January 2000, resulting in 19 fatalities. The disaster response personnel comprised 600 persons from 11 different organisations [9]. Recently, following the terrorist attacks on 22 July 2011, the rescue operation was one of the largest ever undertaken in Norway since World War II. The massive rescue efforts involved a number of agencies from various districts [10].

Analyses of the responses to major incidents nationally and internationally show that certain aspects appear to present particular challenges with regard to interdisciplinary rescue efforts: sufficient training, communication, patient logistics (triage, treatment and transport) and access to equipment [6,11-16]. In this study we wished to map out these elements, as well as the experience and how Norwegian rescue workers think major incident preparedness and on-site accident management function.

## Methods

**Figure 1:**
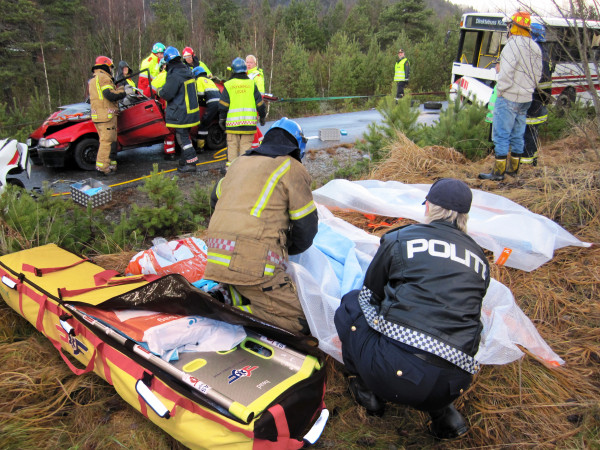
Interdisciplinary cooperation during a TAS course.

In the absence of a civilian Norwegian standard for interdisciplinary rescue operations, the Norwegian Air Ambulance Foundation has developed a course concept for interdisciplinary medical-emergency cooperation (TAS). The TAS courses are offered to the municipalities on a voluntary basis free of charge. The municipalities that choose to participate select the rescue services that will participate and these services select their participants. Since 1998, more than 20,500 persons from various rescue services have attended more than 680 training courses. Personnel from the police, fire departments, salvage services as well as the health services have learned about matters including disaster triage (TAS triage) and structured patient evacuation (OPEN) in simulated disasters within their own local communities (cf. Figure 1).

Participants in a total of 45 out of 49 interdisciplinary medical-emergency cooperation (TAS) courses arranged in 2010 were requested to participate in a survey prior to the start of the courses. Course participants who provided informed consent responded to a questionnaire with items related to their background, their level of experience and access to rescue equipment with special emphasis on triage and stretchers. Furthermore, the rescue workers were asked to indicate how they think certain aspects of on-site work during an accident function and how they think preparedness for a major incident is, using a Likert scale ranging from 1 (doesn’t work) to 7 (works excellently). In the questionnaire rural was defined as a region with fewer than 10,000 inhabitants.

The data were coded in an Excel spreadsheet (Microsoft Corp., USA) and analysed in STATA/SE 11.1 (Statacorp, USA). STROBE (STrengthening the Reporting of OBservational studies in Epidemiology) guidelines for observational research were used in the preparation of the manuscript [17]. The Regional Committee for Medical and Health Research Ethics concluded that the project did not fall under the committee’s mandate (2009/1391a). The Norwegian Social Science Data Service concluded that the study complied with the requirements of the Personal Data Act (22993/2/GRH).

Data are displayed as percentages of all those who answered the question. The number of non-responses is shown in the tables. Responses on the Likert scale are presented as medians, and the range is given in brackets.

## Results

**Figure 2:**
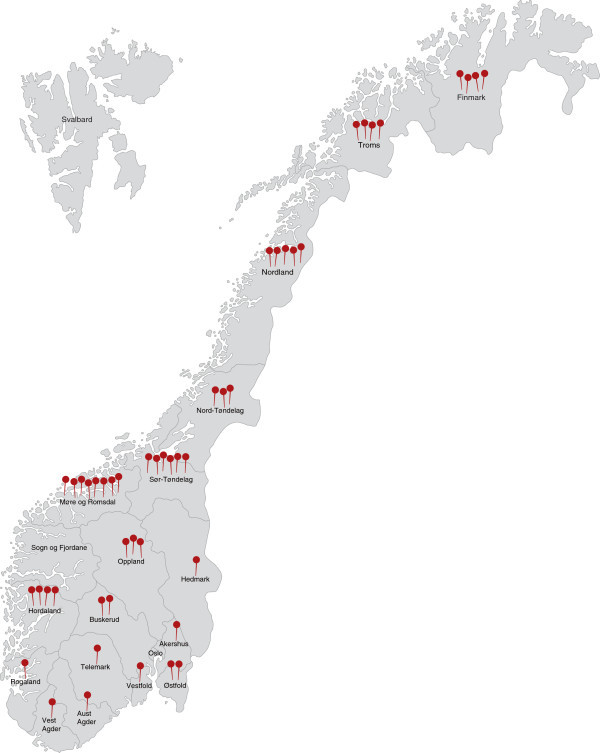
Map of Norway with an overview of implemented TAS courses in 2010.

A total of 999 persons responded to the questionnaire. Of a total of 430 Norwegian municipalities (in 2010) and 19 counties, altogether 50 and 17 respectively are represented in this study (Figure 2). Those who did not enter any profession (*n* = 2) or ticked the box for vehicle salvage worker (*n* = 43) or “other” (*n* = 36) were excluded from the analysis. Data on the background of the participants are provided in Table 1.

**Table 1:** **Participants**’ **backgrounds**

	Age*	**Gender, % men**	Number of years of experience*	Number of major incidents where participated last 10 years*	Number of exercises where participated last 3 years*	**Area, % rural**	**Region, % coastal**
**Health 34.1%*(n*** = **313)**	**36** (15–65) <2>	**571.%***n* = 177 <3>	**7** (0–38) <29>	**1** (0–15) <96>	**1** (0–10) <51>	**64**.**4%***n* = 201 <1>	**60**.**6%***n* = 186 <6>
**Fire 54.1%*(n*** = **497)**	**43** (20–64) <9>	**99**.**4%***n* = 493 <1>	**11** (0–41) <10>	**1** (0–50) <173>	**1** (0–20) <113>	**70**.**7%***n* = 350 <2>	**57**.**2%***n* = 281 <6>
**Police 11.8%*(n*** = **108)**	**40** (22. 59) <0>	**83**.**3%***n* = 90 <0>	**15** (0–39) <4>	**4** (0–25) <23>	**1** (0–10) <10>	**48**.**2%***n* = 52 <0>	**50**.**9%***n* = 55 <0>

*Median in bold, range in brackets.

The number of non-respondents to each question is given in < >.

Results from questions regarding the participants’ experience of major incident preparedness and on-site management are provided in Table 2. Training for disaster response has a median score of 5 among health personnel and 4 among firefighters and police officers. Questions regarding on-site management and interdisciplinary cooperation both return a median score of 5 from all groups of professions. The functioning of the on-site general practitioner (GP) is ranked somewhat lower (4 among health personnel and police officers, 5 among fire-fighting personnel).

**Table 2:** How the participants think preparedness and major incident management function

**How do you think:**					
	**Exercises for operations in case of major incidents function?**	**Your service**’**s preparedness for operations in case of major incidents is?**	**The management at the scene of the incident functions?**	**The interdisciplinary cooperation at the scene of the incident functions?**	**The GP on call at the scene of the incident functions?**
**Health 34.1%*(n*** = **313)**	**5** (1–7) <24>	**5** (1–7) <29>	**5** (2–7) <30>	**5** (3–7) <29>	**4** (1–7) <35>
**Fire 54.1%*(n*** = **497)**	**4** (1–7) <25>	**5** (1–7) <29>	**5** (1–7) <17>	**5** (1–7) <22>	**5** (1–7) <49>
**Police 11.8%*(n*** = **108)**	**4** (1–7) <2>	**5** (1–7) <3>	**5** (2–7) <3>	**5** (3–7) <2>	**4** (1–7) <13>

Median in bold, range in brackets.

The number of non-respondents is given in < >.

Results pertaining to access to equipment for triage marking and emergency stretchers are provided in Table 3. The major proportion of each of these professions was unable to say how long it would take to bring emergency stretchers to the scene of the incident (45.1%, 44.7% and 60.4% for health workers, firefighters and police officers respectively). The majority of each profession (47.9%, 41.7% and 53.5% for health workers, firefighters and police officers respectively) didn’t know if the availability of emergency stretchers would serve as a complicating factor during major incidents in their area.

**Table 3:** Equipment available at the scene of the incident

		**Does your service have a system for sorting and prioritising patients (triage) involved in a major incident? (n)**	**Is marking equipment for sorting of patients (triage) available in your service? (n)**	**Is a stockpile of stretchers available in your area? (n)**
**HEALTH**	**Yes**	**77**.**5%** (231)	**56**.**3%** (166)	**51**.**2%** (153)
**34.1%**	**No**	**7**.**4%** (22)	**28**.**8%** (85)	**13**.**4%** (40)
***n*** = **313**	**Don**’**t know**	**15**.**1%** (45)	**14**.**9%** (44)	**35**.**5%** (106)
	**No response**, ***n***	**15**	**18**	**14**
**FIRE**	**Yes**	**31**.**8%** (153)	**12**.**4%** (58)	**42**.**2%** (202)
**54.1%**	**No**	**30**.**4%** (146)	**48**.**0%** (225)	**20**.**0%** (96)
***n*** = **497**	**Don**’**t know**	**37**.**8%** (182)	**39**.**7%** (186)	**37**.**8%** (181)
	**No response**, ***n***	**16**	**28**	**18**
**POLICE**	**Yes**	**29**.**8%** (31)	**24**.**5%** (26)	**33**.**3%** (35)
**11.8%**	**No**	**42**.**3%** (44)	**51**.**9%** (55)	**17**.**1%** (18)
***n*** = **108**	**Don**’**t know**	**27**.**9%** (29)	**23**.**6%** (25)	**49**.**5%** (52)
	**No response**, ***n***	**4**	**2**	**3**

Percentages given as proportions of responses.

## Discussion

Norwegian rescue workers find that major incident preparedness and on-site work function well. The range in the answers emphasises the heterogeneity among Norwegian rescue workers. Even though major incidents occur only rarely, such events impose great demands on rescue workers in terms of their competence. At the same time, society expects the rescue services to provide an immediate and effective response. Thus training for such events is especially important. All the disciplines included in our survey report that they perceive a need for more training for such interdisciplinary rescue operations. Systematisation and team-based training in trauma care has previously been documented to have beneficial in-hospital effects [18]. Trials of a similar model have been undertaken in the municipal health services and show that the participants subsequently have better confidence in their own roles [19]. Moreover, it has been demonstrated that the sense of coping increases in pace with the amount of training [20]. One of the conclusions drawn after the Sleipner accident was that realistic exercises ought to be undertaken in which focus should be placed on cooperation with other agencies [16]. In Norway there is no common training of on-site commanders (health, fire and police); we believe this is a complicating factor for interdisciplinary collaboration. Such training is expensive, and it is important to establish national guidelines that can ensure that such training is implemented, publicly funded and meets a minimum of standards.

Rescue workers find that evacuation of patients presents challenges, in spite of good availability of resources [11,12]. The World Health Organisation regards triage and evacuation of patients as essential skills in pre-hospital trauma care [21]. In Norway, however, we have no national standards for major incident triage [22] or for the evacuation of patients. With a diverse geography and varying population density, the time needed before the ambulance can arrive, as well as the skills needed by the ambulance personnel, may vary [23,24]. When major incidents occur in sparsely populated areas, the local fire brigade and police may be on site of a major incident for a longer period of time without health workers [21]. Before the arrival of health resources, these emergency services are expected to initiate life-saving measures. It has been shown that non-health professionals can learn triage and that access to written guidelines, as well some experience, improves triage accuracy [25]. It is therefore of grave concern that only 77.5% of the EMS staff utilised a system for triage. Further, only 56% of the EMS responders had access to triage tagging equipment, indicating a need for standardised solutions for triage and marking of priority [22]. Experience from the terrorist bombings in London showed that advanced pre-hospital skills improved triage accuracy [13]; however before such skills are available, simple concepts may improve the precision and speed of the triage process [26], as well as rationalise the evacuation of patients and the interdisciplinary cooperation at the scene of the accident [27]. The inquiry after the avalanche disaster in Vassdalen recommended the introduction of more stringent communication routines, better availability of rescue dogs and better avalanche response equipment [28]. The importance of access to sufficient equipment was also underscored after the incidents on 22 July 2011, when it was described how the emergency stretchers used during the rescue operation helped improve patient logistics [10].

This study shows that the function of the on-site GP has potential for improvement. On-call GPs provide emergency primary health-care services to patients arriving at community-based casualty clinics. In addition, they make house calls and also to various degrees attend patients on site together with the ambulance services outside the larger cities. In many areas the Emergency Medical Communication Centres (EMCC) are instructed to alert all red responses simultaneously to ambulance units and the GP on-call [29]. Recent reports have found decreasing GP involvement in these responses [24,30]. The on-site GPs may have varying experience in emergency incidents [31], and some difficulties have been reported with regard to establishing contact with them for emergency assignments [24]. No formal competence in emergency medicine beyond medical school is required for GPs on- call and experience with emergency procedures remains low [32]. The Norwegian Centre for Emergency Primary Health Care is seeking to establish required national professional standards and to increase attendance by GPs in basic, continuing and further training [33]. We believe that national standards for on-call GPs and attendance in interdisciplinary training may help improve their function at the scene of the incident.

The study of the Norwegian emergency rescue services describes the main groups of rescue workers from various parts of the country. Oslo and Sogn og Fjordane counties and the Svalbard archipelago are not represented in the study as these counties did not apply for a course in the study period, but we assume that this will only have a marginal effect on the general results. The participants were included in local rescue preparedness plans during the course period, and alarms during the course caused unexpected absence. We therefore have no exact information on the number of non-respondents. This risk remained unchanged during the entire training period for all course venues, and we do not believe that this has caused a systematic bias in the results. A limitation of this study is that it relied on self-reported variables, although they may vary in accuracy [34].

A further limitation of this study is the lack of uniform data definitions in the field. The Major Incident Medical Management and Support (MIMMS) definition [6] of major incidents was used in the survey. However it allows room for doubt as to what exactly each individual member of the rescue personnel perceives as a major incident. The definition of rural used in the survey was defined as less than 10,000 inhabitants and the participants were the ones who responded to whether their service is rural or urban. We did not check these data to our definition. Furthermore, there are no uniform operational criteria defining rural areas and studies on rural trauma use a wide range of definitions [35]. We found that the data collected was not collected in a manner that allows us to stratify data as urban or rural with great certainty. We see this as an opportunity missed and would do this differently in a future study. In a major incident both rural and urban services may respond and we believe therefore it is important to focus on establishing national standards for major incident response.

## Conclusions

Rescue personnel find major incident preparedness and on-scene multidisciplinary cooperation to function well. Some shortcomings are reported with regard to systems for major incident triage, tagging equipment for triage and knowledge about access to emergency stretchers. The official enquiry report following 22 July 2011 focused especially on the need for updated interdisciplinary emergency plans and better interdisciplinary cooperation [36]. To comply with these challenges, several national standards such as triage, patient evacuation systems and training concepts need to be implemented. Furthermore, emergency workers should regularly attend exercises focusing on interdisciplinary major incident management.

## Competing interests

All authors are employees of the Norwegian Air Ambulance Foundation, which offers the TAS course free of charge. We have no financial interest, only an intellectual interest in disseminating information about this training course. TV is employed as a consultant for LESS, which is a Norwegian manufacturer of emergency stretchers.

## Authors’ contributions

MR, AJK, JEA and TV conceived the idea and designed the study. JEA and TV collected the data. SF analysed the data. All authors were involved in writing the manuscript and approved the final version.

## Authors’ information

Sabina Fattah (born 1983) is an MD and a PhD fellow in the Norwegian Air Ambulance Foundation/University of Tromsø, focusing on reporting from major incidents. She has experience as a stand-in on-call GP in Hammerfest municipality and as a doctor at the Oslo municipality emergency ward centre.Jan Einar Andersen (born 1962) is a development consultant/project director for the interdisciplinary medical-emergency cooperation, R&D department, Norwegian Air Ambulance Foundation. He is trained as a paramedic and has previously been employed in the ambulance services.

Trond Vigerust (born 1963) is an HCM/rescue paramedic for the Norwegian Air Ambulance and responsible for the field of interdisciplinary medical-emergency cooperation, R&D department, Norwegian Air Ambulance Foundation. Andreas J. Krüger (born 1975) is a resident doctor at the Department of Anaesthesia and Acute Care, St Olavs Hospital, Norway. He is also a PhD fellow in the Norwegian Air Ambulance Foundation/Norwegian University of Science and Technology, focusing on ambulance services staffed by doctors. He has previously been employed by the rescue helicopter services.

Marius Rehn (born 1974) is a resident doctor at the Department of Anaesthesia and Intensive care, Akershus University Hospital, Lørenskog, Norway. He has a Ph.D. in prehospital trauma triage and works as a senior researcher in the Norwegian Air ambulance Foundation. He has previously been employed in the helicopter rescue service.
